# Proteome evaluation of homolog abundance patterns in *Arachis hypogaea* cv. Tifrunner

**DOI:** 10.1186/s13007-022-00840-y

**Published:** 2022-01-13

**Authors:** Zhenquan Duan, Yongli Zhang, Tian Zhang, Mingwei Chen, Hui Song

**Affiliations:** 1 Grassland Agri-Husbandry Research Center, College of Grassland Science, Qingdao Agricultural University, Qingdao, China; 2 College of Animal Science and Technology, Qingdao Agricultural University, Qingdao, China

**Keywords:** Abundance divergence, Homeolog, Paralog, Peanut

## Abstract

**Background:**

Cultivated peanut (*Arachis hypogaea*, AABB genome), an allotetraploid from a cross between *A. duranensis* (AA genome) and *A. ipaensis* (BB genome), is an important oil and protein crop with released genome and RNA-seq sequence datasets. These datasets provide the molecular foundation for studying gene expression and evolutionary patterns. However, there are no reports on the proteomic data of *A. hypogaea* cv. Tifrunner, which limits understanding of its gene function and protein level evolution.

**Results:**

This study sequenced the *A. hypogaea* cv. Tifrunner leaf and root proteome using the tandem mass tag technology. A total of 4803 abundant proteins were identified. The 364 differentially abundant proteins were estimated by comparing protein abundances between leaf and root proteomes. The differentially abundant proteins enriched the photosystem process. The number of biased abundant homeologs between the two sub-genomes A (87 homeologs in leaf and root) and B (69 and 68 homeologs in leaf and root, respectively) was not significantly different. However, homeologous proteins with biased abundances in different sub-genomes enriched different biological processes. In the leaf, homeologs biased to sub-genome A enriched biosynthetic and metabolic process, while homeologs biased to sub-genome B enriched iron ion homeostasis process. In the root, homeologs with biased abundance in sub-genome A enriched inorganic biosynthesis and metabolism process, while homeologs with biased abundance in sub-genome B enriched organic biosynthesis and metabolism process. Purifying selection mainly acted on paralogs and homeologs. The selective pressure values were negatively correlated with paralogous protein abundance. About 77.42% (24/31) homeologous and 80% (48/60) paralogous protein pairs had asymmetric abundance, and several protein pairs had conserved abundances in the leaf and root tissues.

**Conclusions:**

This study sequenced the proteome of *A. hypogaea* cv. Tifrunner using the leaf and root tissues. Differentially abundant proteins were identified, and revealed functions. Paralog abundance divergence and homeolog bias abundance was elucidated. These results indicate that divergent abundance caused retention of homologs in *A. hypogaea* cv. Tifrunner.

**Supplementary Information:**

The online version contains supplementary material available at 10.1186/s13007-022-00840-y.

## Introduction

Cultivated peanut (*Arachis hypogaea*), an important oil and protein crop, is an allotetraploid from a cross between *A. duranensis* and *A. ipaensis* [1–3]. *Arachis* spp. genomes, including *A. duranensis*, *A. ipaensis*, and *A. hypogaea* cv. Tifrunner have been sequenced [1, 2]. *Arachis hypogaea* cv. Tifrunner is highly resistant to tomato spotted wilt virus and moderately resistant to early and late leaf spot bacteria [4]. The *A. hypogaea* cv. Tifrunner genome has been used for quantitative trait locus analyses and RNA-seq assemblies [5–8]. In 2016, RNA-seq datasets from 22 different tissues of *A. hypogaea* cv. Tifrunner were sequenced under normal growth conditions, and the cleaned raw data was released on PeanutBase [9, 10]. However, there are no proteomic datasets for *A. hypogaea* cv. Tifrunner, which limits the understanding of its gene functions.

Homeolog expression bias is an important feature in polyploidy [11, 12]. *Arabidopsis suecica* (AATT genome) derives from crossing *Arabidopsis thaliana* (AA genome) and *Arabidopsis arenosa* (TT genome) [13]. An RNA-seq data showed that *A. suecica* preferentially expressed homeologous genes towards sub-genome T [14]. Nevertheless, different results in the *Gossypium hirsutum* (AADD genome), an allopolyploid from a cross of *Gossypium arboretum* (AA genome) and *Gossypium raimondii* (DD genome), is a crucial model for homeologous expression bias [11, 12, 15, 16]. The homeologs from sub-genome A had biased expression in diploid hybrids and natural allopolyploids, but the expression was reversed in synthetic allopolyploids [16]. However, homeologs from reproductive tissues of cultivated peanut, including *A. hypogaea* cv. Shitouqi, *A. hypogaea* cv. Tifrunner, and *A. hypogaea* cv. Fuhuasheng, had biased expression in sub-genome B [2, 17, 18]. To date, no study has analyzed homeolog bias using proteomic data. Therefore, this study sequenced the *A. hypogaea* cv. Tifrunner proteome from leaf and root tissues using the tandem mass tag technology. The results revealed proteome level of homolog (including paralogs and homeologs) abundance patterns, providing insights into homolog expression.

## Methods

### Plant materials

Sterile *A. hypogaea* cv. Tifrunner seeds were cultured on sterile, wet Petri dishes at 28 °C in the dark. When the first cotyledon expanded, the germinated plants were transferred into Hoagland solution and continually cultured at 28 °C under 16 h light/8 h dark cycles. Leaf (containing lateral stem leaf, mainstem leaf and seeding leaf) and root tissues were collected from three biological replicates, and samples were stored at − 80 °C for further use.

### Proteome sequencing and assemble

The samples were mixed with 8 M Urea/100 mM Tris-Cl and treated with water bath sonication for 10 min in ice-water bath, and centrifuged for 15 min at 12,000*g*. After centrifugation, the supernatant was subjected to a reduction reaction (10 mM DTT, 37 °C for 1 h), followed by an alkylation reaction (40 mM iodoacetamide, room temperature/dark place for 30 min). The protein concentration was measured by the Bradford method [19]. Total protein was released using a lysis buffer (2.5% SDS, 100 mM Tris-Cl, pH 8.0), and the protein was digested in trypsin at 37 °C. The digested protein was sequenced and quantified using TMT technology. Peptides were TMT-labeled following the manufacturer’s instructions (Thermo Fisher Scientific, MA, USA). Briefly, peptides were reconstituted in TMT reagent buffer (Thermo Fisher Scientific, MA, USA), and the samples were separately labeled with different TMT-labeling reagents. The TMT-labeled samples were mixed and desalted using Sep-Pak C18 (Thermo Fisher Scientific, MA, USA). The complex mixture was fractionated using high pH reverse phase chromatography and combined into 15 fractions. Each fraction was vacuum-dried and stored at − 80 °C until mass spectrometry (MS) analysis.

LC–MS/MS datasets were produced by the Q Exactive HF-X mass spectrometer (Thermo Fisher Scientific, MA, USA) and Easy-nLC 1000 system (Thermo Fisher Scientific, MA, USA). The peptides were loaded through an auto-sampler and separated in a C18 analytical column (50 μm × 15 cm, C18, 2 μm, 100 Å). The mobile phases A (0.1% formic acid) and B (80% ACN, 0.1% formic acid) were used to establish the separation gradient.

The MaxQuant software (v1.66) estimated the MS raw data using the Andromeda search engine. The *A. hypogaea* cv. Tifrunner primary proteins were used to search the proteome database. Proteins denoted as decoy hits, contaminants, or only identified by site were removed. The remaining hits were used for further quantification analysis. Both proteins and peptides were filtered at 1% FDR. The raw datasets were deposited in the ProteomeXchange Consortium (http://proteomecentral.proteomexchange.org) via the iProX partner repository [20] with the dataset identifier PXD027553.

### Differentially abundant proteins

Differential abundance was calculated between leaf and root tissues using protein quantification (abundance). The leaf and root average protein abundance was estimated using three biological replicates. The differential abundance was estimated by log_2_(average abundance in leaf/average abundance in root). Proteins with log_2_(foldchange) was larger than two or less than negative two at *p* ≤ 0.05 significance level using *t*-test, and they were considered differentially abundant.

GO and KEGG enrichment analyses were performed to reveal differential abundance in protein function. The GO enrichment was performed using the eggNOG-mapper tool on differentially expressed *A. hypogaea* cv. Tifrunner protein sequences. The same differentially expressed protein sequences were uploaded to the KEGG database (http://www.genome.jp/tools/kaas) for KEGG annotation. The Fisher test was used for statistical analysis.

### Homologous protein abundance

Paralogs are genes produced via gene duplication within a genome. Homeologs are genes formed by hybridization or polyploidization but not through gene duplication events. Paralogs and homeologs were identified in *A. hypogaea* cv. Tifrunner to reveal proteome-level homolog abundance. The *A. hypogaea* cv. Tifrunner homeologs were inferred from the *A. duranensis* and *A. ipaensis* genome sequences. The *A. duranensis*, *A. ipaensis*, and *A. hypogaea* cv. Tifrunner genome sequences were downloaded from the Peanutbase database (https://www.peanutbase.org/) [1, 2, 9]. Homologs were identified using the previously described BLAST-based method [10, 21, 22]. The following thresholds were used: (1) The alignment sequence length is > 80% in each sequence. (2) the sequence similarity is > 80%, and (3) the E-value is 1E−10.

This study also analyzed the paralog divergence and homeolog abundance bias. A paralogous pair was considered abundantly divergent when the absolute value of log_2_(copy1 abundance/copy2 abundance) was larger than two in the paralogous pair at *p* ≤ 0.05 significance level using the *t*-test. For log_2_(sub-genome A abundance/sub-genome B abundance) values was larger than two or less than negative two in a homeologous pair at *p* ≤ 0.05 significance level on the *t*-test, the homeologous pair was considered biased for sub-genomes A or B.

To estimate the selective pressures of paralogs and homeologs, the homologous protein pairs were aligned using the MAFFT program [23] and converted to CDS using the PAL2NAL program [24]. The nonsynonymous substitutions per nonsynonymous site (*K*_*a*_) and synonymous substitutions per synonymous site (*K*_*s*_) were estimated. The sequences with low sequence divergence (*K*_*a*_*/K*_*s*_ value < 0.001 or *K*_*a*_ = 0) were excluded because low sequence divergence causes unknown results. Neutrality, positive selection, and purifying selection were indicated by *K*_a_/*K*_s_ = 1, *K*_a_/*K*_s_ > 1, and *K*_a_/*K*_s_ < 1, respectively.

## Results

### Evaluation of proteomic sequencing

Contamination is a major setback during protein sampling, transportation, and sequencing. Therefore, this study examined all proteome sequences and confirmed purity (Additional file 1: Fig. S1a). Total protein was digested using trypsin (hydration C-terminal end of arginine and lysine), but several arginine and lysine residues were modified after translation. The modified amino acids were indigestible. Most proteins were digested totally, but a few had missed cleavage (Additional file 1: Fig. S1b). The average peptide weight was 110 Da, and the peptide length was 7 to 27 amino acids using the MS analysis (Additional file 1: Fig. S1c). The distribution of mass deviation (effective peptide weight/expected peptide weight) was − 4 to 4 (Additional file 1: Fig. S1d), consistent with the standard, − 10 to 10. Several proteins lacking during the proteome sequencing of leaf and root tissues could not be quantified (Additional file 1: Fig. S1e). The miss rate was 2.53% and 2.61% in the three biological repetitions between leaf and root tissues. The leaf and root tissues were not statistically different (Mann–Whitney test, *p* > 0.05), indicating that missed data did not significantly affect differential abundance. Consequently, the proteome sequencing data were used for further analyses.

### Protein quantification and differentially abundant protein functional annotation

**Fig. 1:**
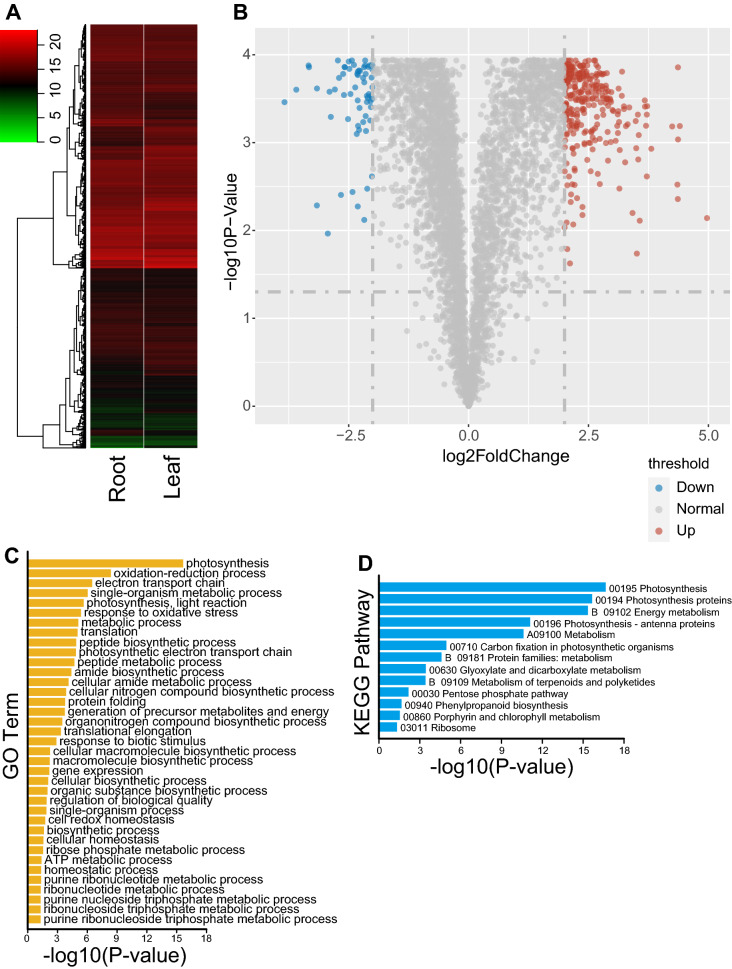
The proteomic abundance of leaf and root tissues. **A** A total of 4803 abundant proteins in the leaf and root tissues. **B** A total of 364 differentially abundant proteins between leaf and root tissues, with 300 up-regulated and 64 down-regulated proteins, respectively. **C** Gene ontology (GO) enrichment analyses using differentially abundant proteins. The GO terms of the biological process were listed. **D** Kyoto encyclopedia of genes and genomes (KEGG) enrichment analyses using differentially abundant proteins

A total of 34,547 peptides (19,257 unique peptides) were identified in the leaf and root tissues, translating to 4803 proteins via the primary transcript CDS (Fig. 1A and Additional file 6: Table S1). A correlation analysis showed that the protein abundance patterns were similar across the three-leaf and root biological replicates (Additional file 1: Fig. S1f). There were 364 differentially abundant proteins between leaf and root tissues, including 300 up-regulated and 64 down-regulated proteins (Fig. 1B).

The GO enrichment results revealed that differentially abundant proteins were preferentially involved in the photosystem process (Fig. 1C). Meanwhile, the KEGG results showed that differentially abundant proteins were enriched for photosynthesis (Fig. 1D). These results indicate that differentially abundant proteins correlated with the environmental changes between leaf and root tissues. The leaf and root are distinct tissues, which leaf is aboveground, and the root is underground.

### Paralog abundance divergence and homeolog abundance bias

**Fig. 2:**
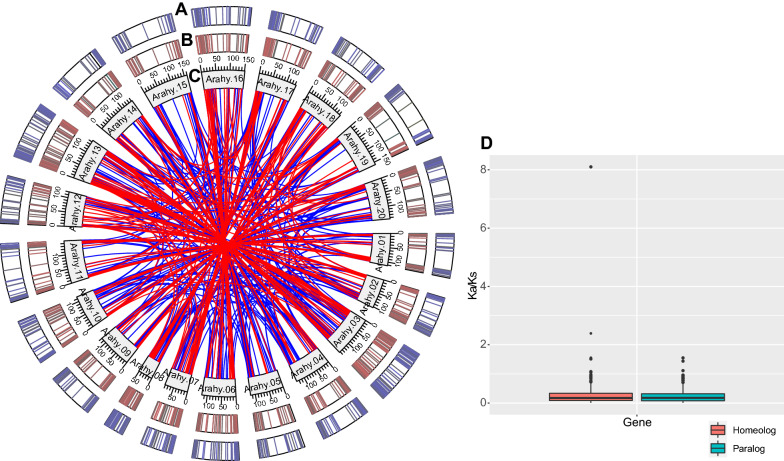
The paralogs and homeologs with protein abundances. **A** The paralogs and homeologs with protein abundances in the root tissue. The deeper color indicates higher protein abundance. **B** The paralogs and homeologs with protein abundance in the leaf tissue. The deeper color indicates higher protein abundance. **C** The chromosome locations of paralogous and homeologous protein pairs. The red line connects a homeologous protein pair, and the blue line connects a paralogous protein pair. **D** The paralogous and homeologous protein pairs underwent purifying selection

The *A. hypogaea* cv. Tifrunner proteome had 336 paralogous and 197 homeologous pairs of abundant proteins (Fig. 2A and Additional file 7: Table S2). The selective pressure results showed that the average *K*_*a*_/*K*_*s*_ value of paralogous and homeologous pairs was 0.24 and 0.31, respectively (Fig. 2B). The results indicate that major purifying selection acted on these proteins. Additionally, the *K*_*a*_/*K*_*s*_ values of the three paralogous and six homeologous pairs were larger than one, indicating that these proteins underwent positive selection (Fig. 2B).

**Fig. 3:**
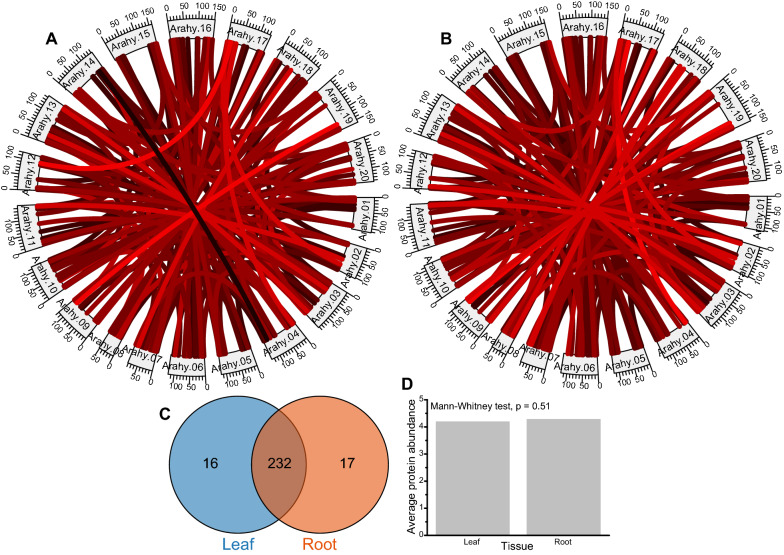
Comparison of paralogous protein abundances between leaf and root tissues. **A** The differential abundance of paralogous pairs in the leaf. The deeper red color indicates higher protein differential abundance. The black color indicates lower protein differential abundance. **B** The differential abundance of paralogous pairs in the root. The deeper red color indicates higher protein differential abundance. The black color indicates lower protein differential abundance. **C** The number of paralogous protein pairs between leaf and root tissues. **D** The average protein abundance between leaf and root tissues in 232 common paralogous protein pairs

**Fig. 4:**
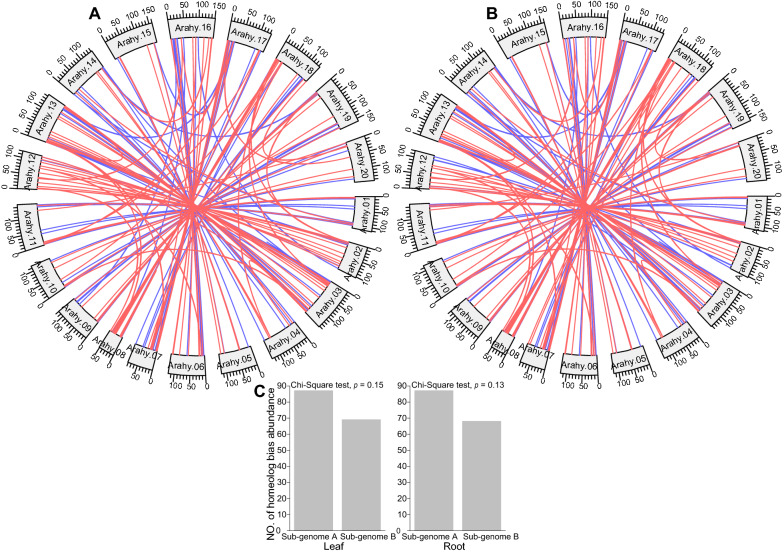
Homeologous protein abundances in the leaf and root tissues. **A** The homeologous abundance bias in the leaf. The red and blue colors indicate homelogous protein abundances of sub-genomes A and B. **B** The homeologous abundance bias in the root. The red and blue colors indicate homeologous protein abundance toward sub-genomes A and B. **C** The number of homeologous protein pairs with bias abundance in the leaf and root tissues

A total of 248 and 249 paralogous pairs were divergently abundant in the leaf and root tissues, respectively (Fig. 3a, B) There were 232 common paralogous pairs between leaf and root tissues (Fig. 3C). However, divergent abundance in the 232 paralogous pairs was not significantly different (Fig. 3D). The results indicate that the paralogous pairs from leaf and root tissues had similar divergent abundances. The same leaf and root tissues had 156 and 155 homeologous pairs with biased abundances (Fig. 4A, B), including 87 homeologous pairs biased to sub-genome A and 69 biased to sub-genome B leaf tissue. In the root, 87 homeologous pairs were biased to sub-genome A, while 68 were biased to sub-genome B. The leaf and root tissue were not significantly different, considering the number of homeologs with biased abundances (Fig. 4C).

GO enrichment analyses were executed in the leaf and root tissue to reveal homeologous protein function. In the leaf, homeologs biased to sub-genome A enriched biosynthetic and metabolic process, while homeologs biased to sub-genome B enriched iron ion homeostasis process (Additional file 2: Fig. S2 and Additional file 3: Fig. S3). In the root, homeologs with biased abundance enriched biosynthetic and metabolic processes (Additional file 4: Fig. S4 and Additional file 5: Fig. S5). However, sub-genomes A and B had different homeologs in the root. Homeologs with biased abundance in sub-genome A enriched inorganic biosynthesis and metabolism process, while homeologs with biased abundance in sub-genome B enriched organic biosynthesis and metabolism process (Additional file 4: Fig. S4 and Additional file 5: Fig. S5). These results indicate that homeologs had a functional bias in the different sub-genomes and tissues.

**Fig. 5:**
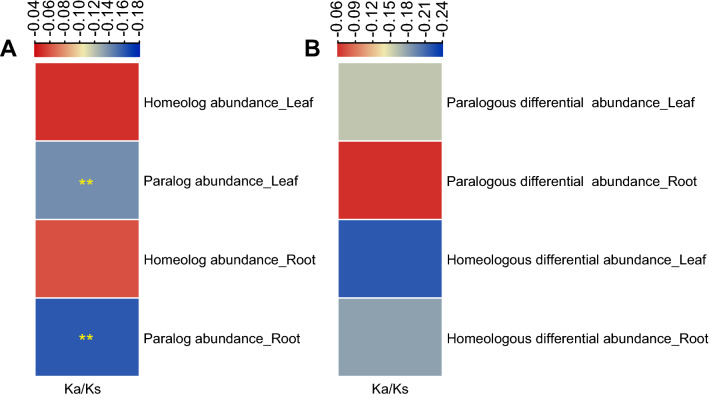
Correlations of paralogous and homeologous protein abundances and selective pressure in the leaf and root tissues. **A** Correlations between paralogous and homeologous protein abundances and selective pressure in the leaf and root tissues. **B** Correlations between paralogous and homeologous differential abundances and selective pressure in the leaf and root tissues. *K*_*a*_/*K*_*s*_, ratios of nonsynonymous substitutions per nonsynonymous site (*K*_*a*_) to synonymous substitutions per synonymous site (*K*_*s*_). **Indicates significant differences at 0.01 using the Mann–Whitney test

The *K*_*a*_/*K*_*s*_ values were not correlated with homeologous protein abundance in the leaf and root tissues but negatively correlated with paralogous protein abundance (Fig. 5A). The correlation between *K*_*a*_/*K*_*s*_ value and protein abundance in paralogs is consistent with the expression rate of sequence evolution anticorrelation model (E-R anticorrelation) [25], where decreasing protein misfolding promotes fitness [26]. Moreover, the *K*_*a*_/*K*_*s*_ values and differential protein abundances in paralogs and homeologs of leaf and root tissues were not correlated (Fig. 5B). These results indicate that selective pressure cannot affect differential protein abundance, consistent with a previous study where *A. duranensis* CDS architectures and differential gene expression were not correlated under drought and nematode stress [27].

### Paralogs and homeologs have differential abundance between leaf and root

**Fig. 6:**
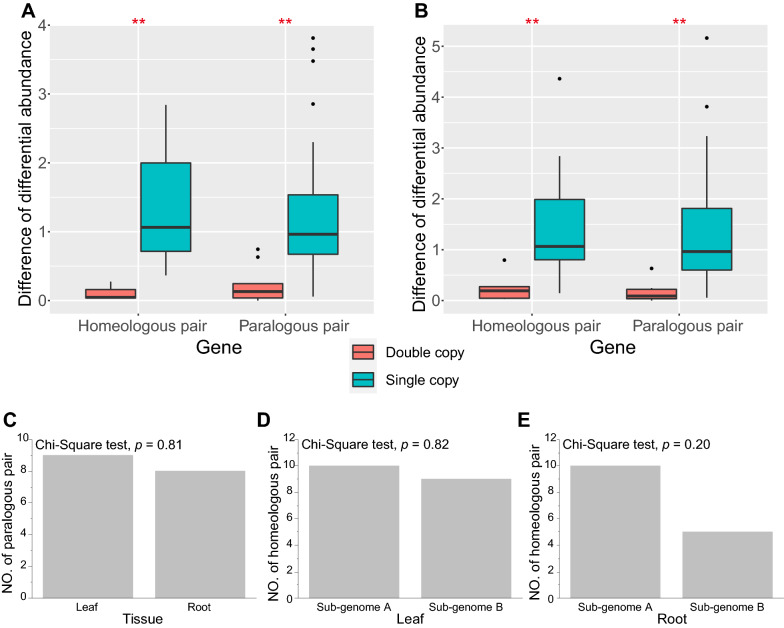
Paralogs and homeologs with differential abundances between leaf and root tissues. **A** Differential abundances of paralogous and homeologous pairs between leaf and root tissues in the leaf. **B** Differential abundances of paralogous and homeologous pairs between leaf and root tissues in the root. **C** The number of paralogous pairs where the two copies were differentially abundant between leaf and root tissues. **D** The number of homeologous pairs where the two copies were abundantly biased in the leaf. **E** The number of homeologous pairs where the two copies were abundantly biased in the root. Double copy indicates that two copies from the paralogous pair and the homeologous pair are differentially abundant. Single copy indicates that one copy from the paralogous pair and the homeologous pair is differentially abundant. **Indicates significant differences at 0.01 using the Mann–Whitney test

This study identified differentially abundant proteins between leaf and root tissues. However, little is known about the abundance patterns of paralogs and homeologs in the differentially abundant proteins. One copy of the 48 paralogous protein pairs was differentially abundant in the leaf and root tissues (Additional file 8: Table S3). Twelve paralogous protein pairs were differentially abundant in the leaf and root tissues (Additional file 8: Table S3). In the leaf and root tissues, 24 homeologous protein pairs had one differentially abundant copy (Additional file 9: Table S4). Seven homeologous protein pairs were differentially abundant in the leaf and root (Additional file 9: Table S4). The two copies from paralogous and homeologous protein pairs had differential abundances, indicating that the two copies had similar gene functions. In contrast, only one copy from paralogous and homeologous protein pairs had differential abundances, indicating that the two copies had potentially divergent functions. Furthermore, the difference value of differential abundance from protein pairs with two differentially abundant copies was less than that of differential abundance from protein pairs with one differentially abundant copy (Fig. 6A, B).

In paralogous proteins, 33 paralogous protein pairs with one differentially abundant copy were divergently abundant in the leaf and root. In addition, eight and nine paralogous protein pairs were divergently abundant in the root and leaf, respectively (Fig. 6C). These results indicate that paralogous protein pairs with differential abundance had similar abundance patterns in the leaf and root tissues. Ten homeologous protein pairs were biased to sub-genome A, and nine gene pairs were biased to sub-genome B in the leaf (Fig. 6D). In the root, five and ten homeologous protein pairs had biased abundance in the root sub-genomes B and A, respectively (Fig. 6E). These results indicate that homeologous protein pairs with differential abundance had similar abundance patterns in the leaf and root tissues.

## Discussion

This work provides a foundation for revealing gene functions and improving molecular breeding of *A. hypogaea* cv. Tifrunner [2]. To date, homeolog expression bias was only elucidated using the RNA-seq analysis of cultivated peanut [2, 10, 17, 18, 28]. However, proteome level homeolog abundance bias in *A. hypogaea* cv. Tifrunner is scarcely known. This study obtained proteomic datasets from leaf and root tissues using the TMT technology and estimated differentially abundant proteins between leaf and root tissues. The paralogous protein abundance divergence and homeologous abundance bias were revealed. The study showed that 364 proteins were differentially abundant between leaf and root tissues. These differentially divergent proteins enriched the photosystem process. Several homeologous proteins had biased abundances in sub-genomes A and B, but the number was not significantly different in the leaf and root tissues. Furthermore, the homeologous biased proteins between sub-genomes A and B had different biological processes. Additionally, paralogous and homeologous proteins were divergently abundant, with potentially functional divergence.

To our knowledge, there are no reported studies on proteomic homeologous abundance bias in *A. hypogaea* cv. Tifrunner. This study identified 156 homeologous protein pairs with biased abundance, yet previous study identified 7404 preferentially expressed homeologous gene pairs [2]. Although the number of homeologs between these two studies is different, both studies showed that the number of biased homeologous pairs between sub-genomes A and B was not significantly different in the leaf and root tissues. Previous studies demonstrated non-significant differences in homeolog expression bias in somatic tissues but reproduction tissues in *A. hypogaea* cv. Tifrunner, *A. hypogaea* cv. Shitouqi, and *A. hypogaea* cv. Fuhuasheng were significantly different [2, 17, 18]. Homeolog expression bias is tissue-specific in cultivated peanuts compared to other allotetraploid plants. Although homeolog expression bias varies between tissues, other allotetraploid plants, such as cotton (*Gossypium hirsutum*), report common expression bias. In *G. hirsutum*, 20–40% of homeologs had biased expression towards sub-genome D, and a few gene pairs were biased toward sub-genome A in 35 different tissues [29]. Additionally, homeologous expression was biased in sub-genome A during the fiber development of cultivated cotton [29].

There are different functions of homeologs between two sub-genomes in *A. hypogaea* cv. Tifrunner [2]. For example, A sub-genome-biased homeologous gene pairs are preferentially involved in mannose metabolic processes, nitrate assimilation, and cell wall assembly, and B sub-genome-biased homeologous gene pairs are enriched in response to biotic stimulus, sucrose transport, and glucan metabolic processes in subterranean peg tip [2]. In maturing pericarp, the A sub-genome-biased homeologous gene pairs were biased toward phosphorylation signal transduction, carbohydrate metabolism, and cell wall biogenesis. In contrast, B sub-genome-biased homeologous gene pairs were enriched for response to biotic stimulus and inorganic ion transport [2]. Additionally, comparisons of genome sequences between *A. monticola* and *A. hypogaea* cv. Tifrunner revealed that domestication mainly affects the sub-genome A of *A. hypogaea* cv. Tifrunner [28]. However, this study showed that homeologous proteins had different biological processes in the two sub-genomes from leaf and root tissues, consistent with Bertioli et al. [2]. These results indicate that homeologs are crucial for functional divergence between the two sub-genomes.

Homologs have functionally divergent gene expression [30–32] due to the WGD that preferentially occurred 55–75 million years ago around the K/Pg boundary [33]. This period involved numerous cataclysmic events causing biological extinctions [34]. The WGDs prevented extinctions of flowering plants [33]. This study showed that most homologous protein pairs had asymmetric abundance, and several homologous protein pairs had conserved abundance between leaf and root tissues. These results provide a clue about asymmetric abundance with potential functions in homologs retention at genome and proteome levels.

## Conclusions

In this study, proteome data were used to reveal homologous abundance patterns in *A. hypogaea* cv. Tifrunner. The results showed there is no difference in the number of biased abundant homeologs between the sub-genomes A and B. However, homeologous proteins enriched different biological processes between the two sub-genomes. Paralogous and homeologous biased abundant divergence in leaf and root tissues.

## Supplementary Information

**Additional file 1: Fig. S1.** Proteome sequencing results of leaf and root tissues by the tandem mass tag technology. A. The protein contaminant information during proteome sequencing. B. The number of missed cleavages. C. The peptide length. D. The distribution of mass deviation. E. The number of non-detected proteins. F. The protein abundance correlation across biological replications.

**Additional file 2: Fig. S2.** The gene ontology enrichment of biased homeologs in the leaf sub-genome A.

**Additional file 3: Fig. S3.** The gene ontology enrichment of biased homeologs in the leaf sub-genome B.

**Additional file 4: Fig. S4.** The gene ontology enrichment of biased homeologs in the root sub-genome A.

**Additional file 5: Fig. S5.** The gene ontology enrichment of biased homeologs in the root sub-genome B.

**Additional file 6: Table S1.** Proteins with abundances were detected using the tandem mass tag technology.

**Additional file 7: Table S2.** The paralogous and homeologous protein pairs with abundances in the leaf and root tissues.

**Additional file 8: Table S3.** The paralogous and homeologous protein pairs with differential abundances in the leaf. The differential abundance was estimated between leaf and root tissues.

**Additional file 9: Table S4.** The paralogous and homeologous protein pairs with differential abundances in the root. The differential abundance was estimated between leaf and root tissues.

## Data Availability

All of the data and materials supporting our research findings are contained in the methods section of the manuscript. Details are provided in the attached additional files.

## Abbreviations

FDR: False discovery rate
GO: Gene ontology
*K*_*a*_: Nonsynonymous substitutions per nonsynonymous site
KEGG: Kyoto encyclopedia of genes and genomes
*K*_*s*_: Synonymous substitutions per synonymous site
MS: Mass spectrometry
TMT: Tandem mass tag
