# Supplementary figures and images for: Proteome evaluation of homolog abundance patterns in Arachis hypogaea cv. Tifrunner
Source: Plant Methods. 2022 Jan 13;18:6. doi: 10.1186/s13007-022-00840-y (PMC8756696; doi:10.1186/s13007-022-00840-y)

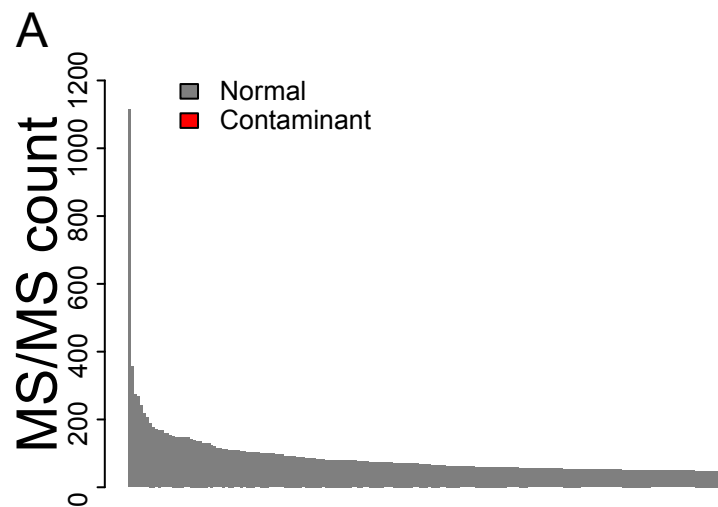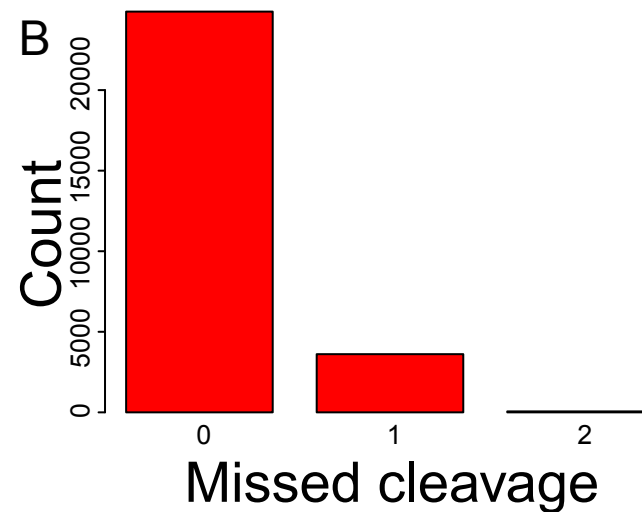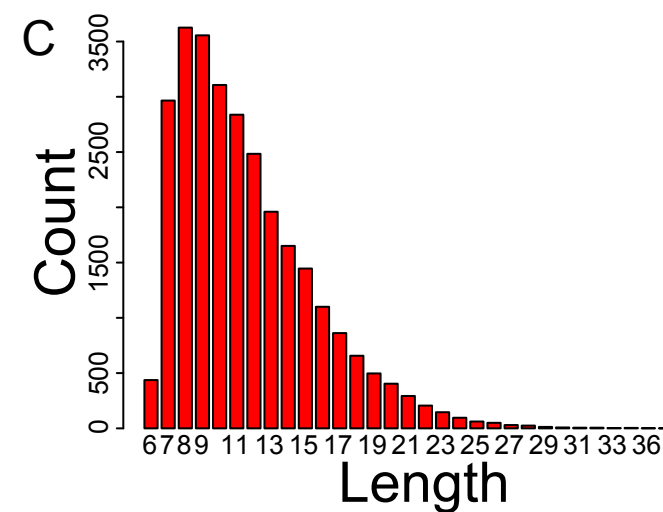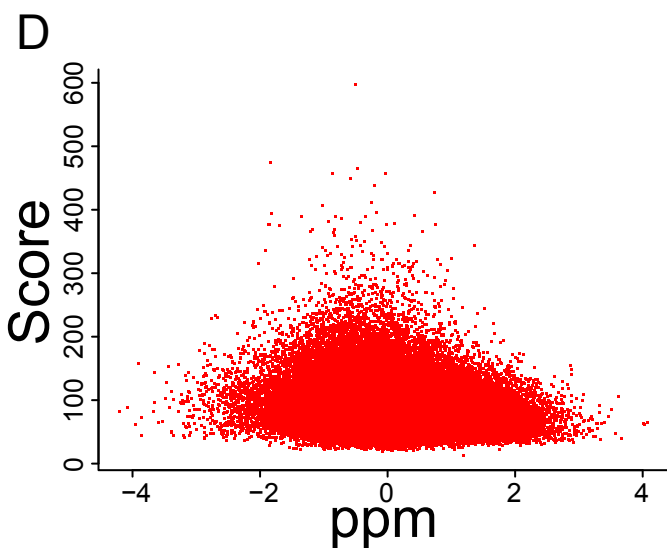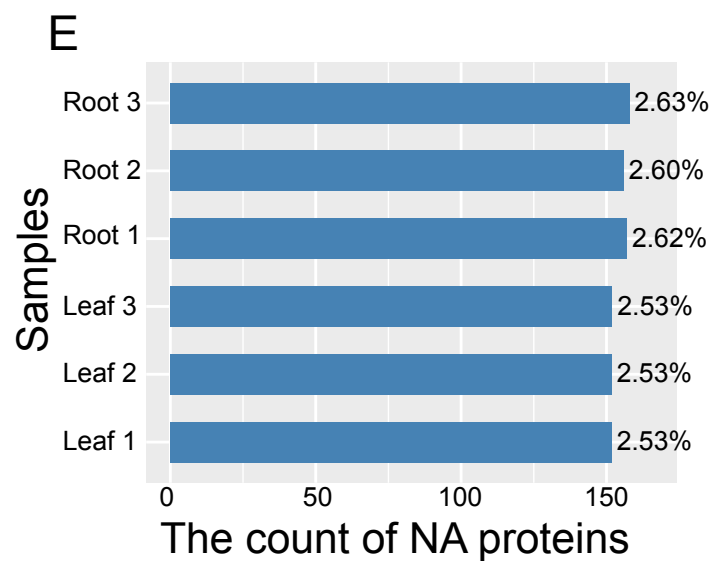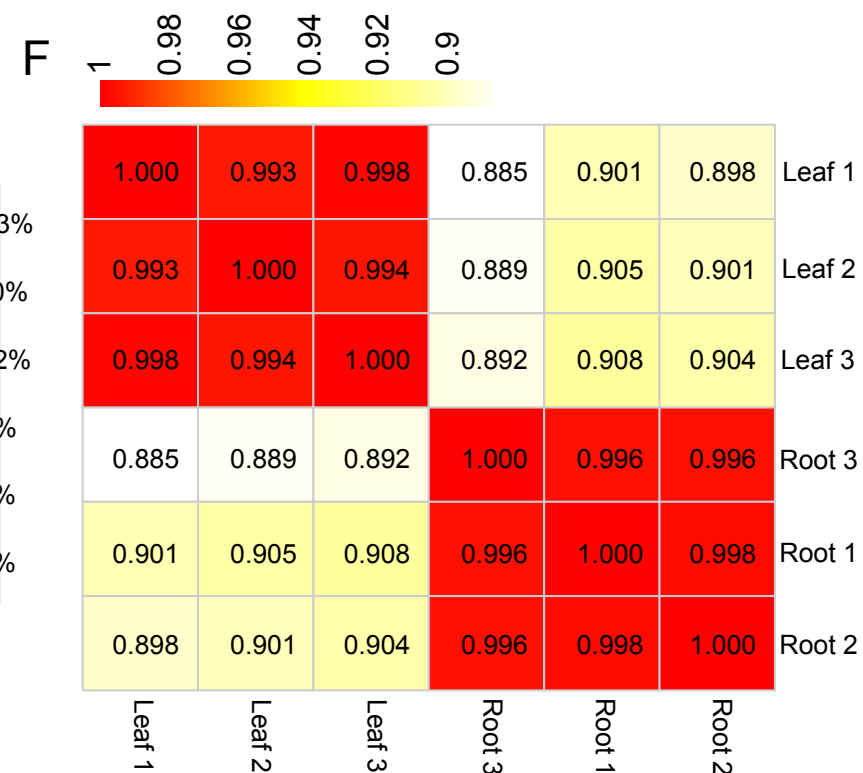

Supplement: Supplementary file 1 — Additional file 1: Fig. S1. Proteome sequencing results of leaf and root tissues by the tandem mass tag technology. A. The protein contaminant information during proteome sequencing. B. The number of missed cleavages. C. The peptide length. D. The distribution of mass deviation. E. The number of non-detected proteins. F. The protein abundance correlation across biological replications. [file 13007_2022_840_MOESM1_ESM.pdf]

GO Term

0.00 1.00 2.00 3.00 4.00 5.00 6.00 7.00 8.00

-log10(P-value)

- Molecular function
- Cellular component
- Biological process

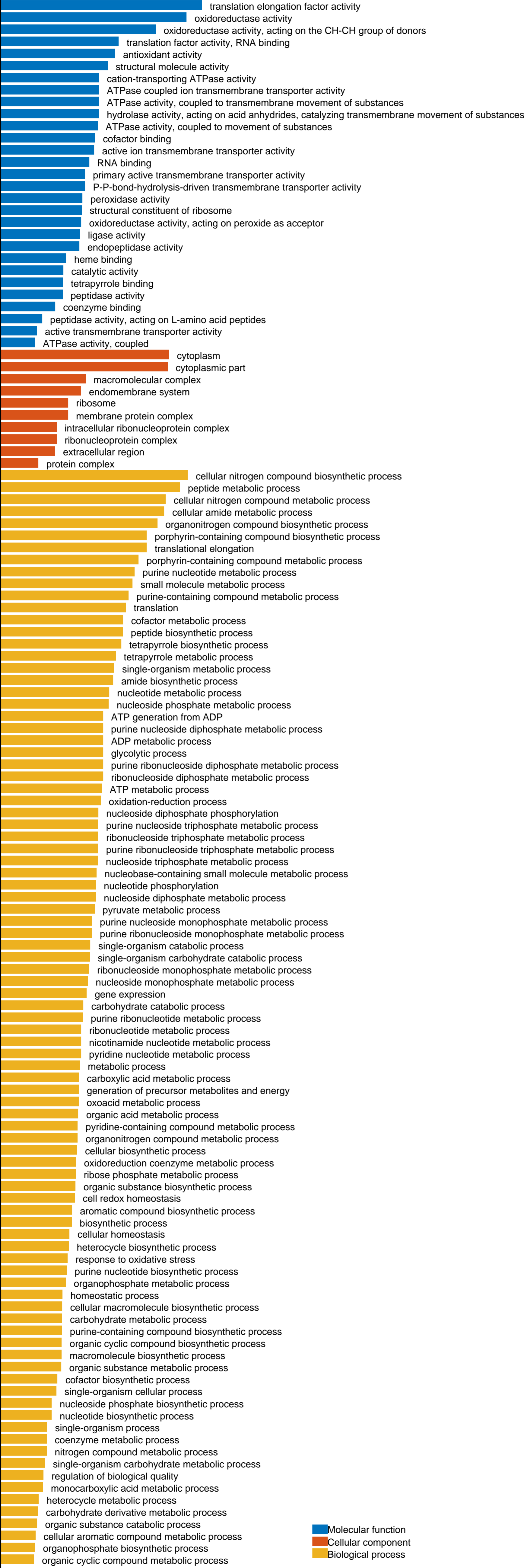

Supplement: Supplementary file 2 — Additional file 2: Fig. S2. The gene ontology enrichment of biased homeologs in the leaf sub-genome A. [file 13007_2022_840_MOESM2_ESM.pdf]

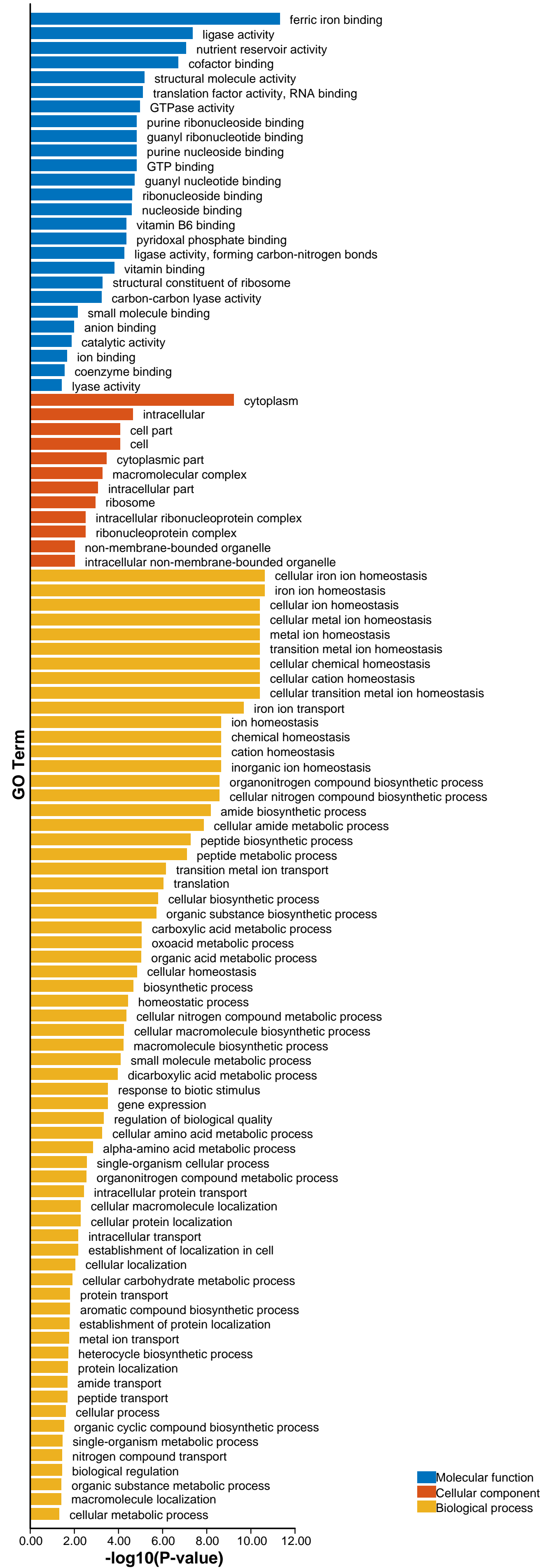

Supplement: Supplementary file 3 — Additional file 3: Fig. S3. The gene ontology enrichment of biased homeologs in the leaf sub-genome B. [file 13007_2022_840_MOESM3_ESM.pdf]

GO Term

0.002.004.006.008.0010.00

-log10(P-value)

Molecular function  
Cellular component  
Biological process

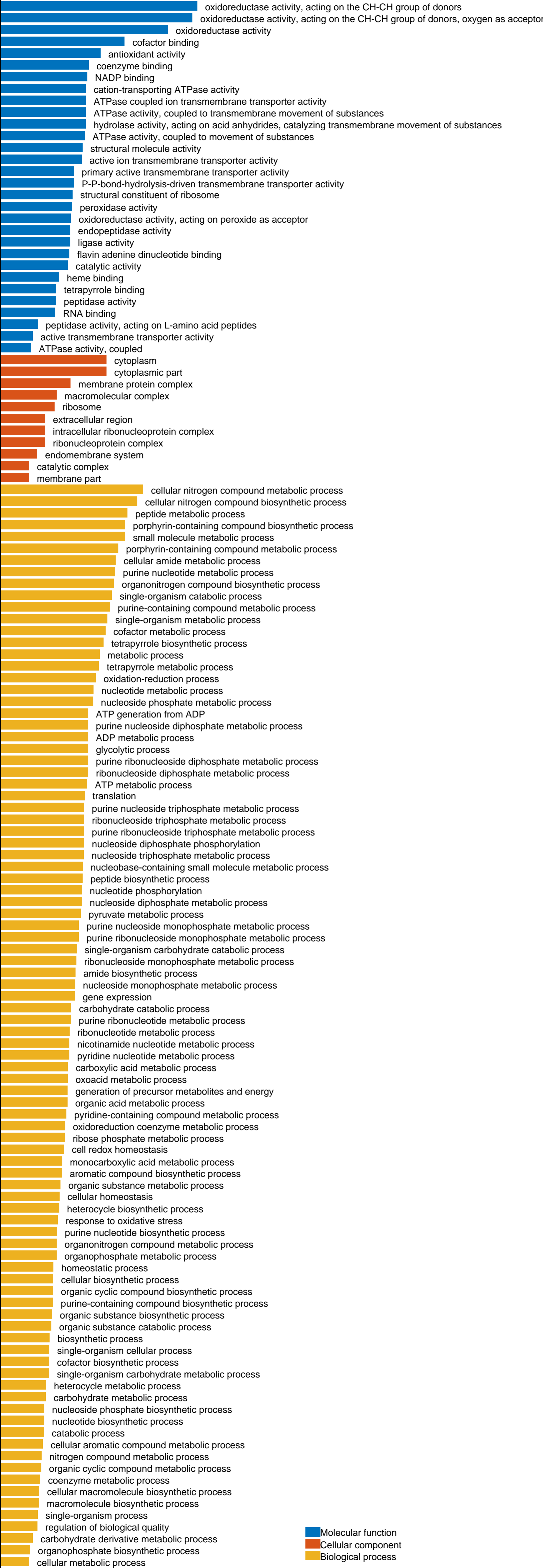

Supplement: Supplementary file 4 — Additional file 4: Fig. S4. The gene ontology enrichment of biased homeologs in the root sub-genome A. [file 13007_2022_840_MOESM4_ESM.pdf]

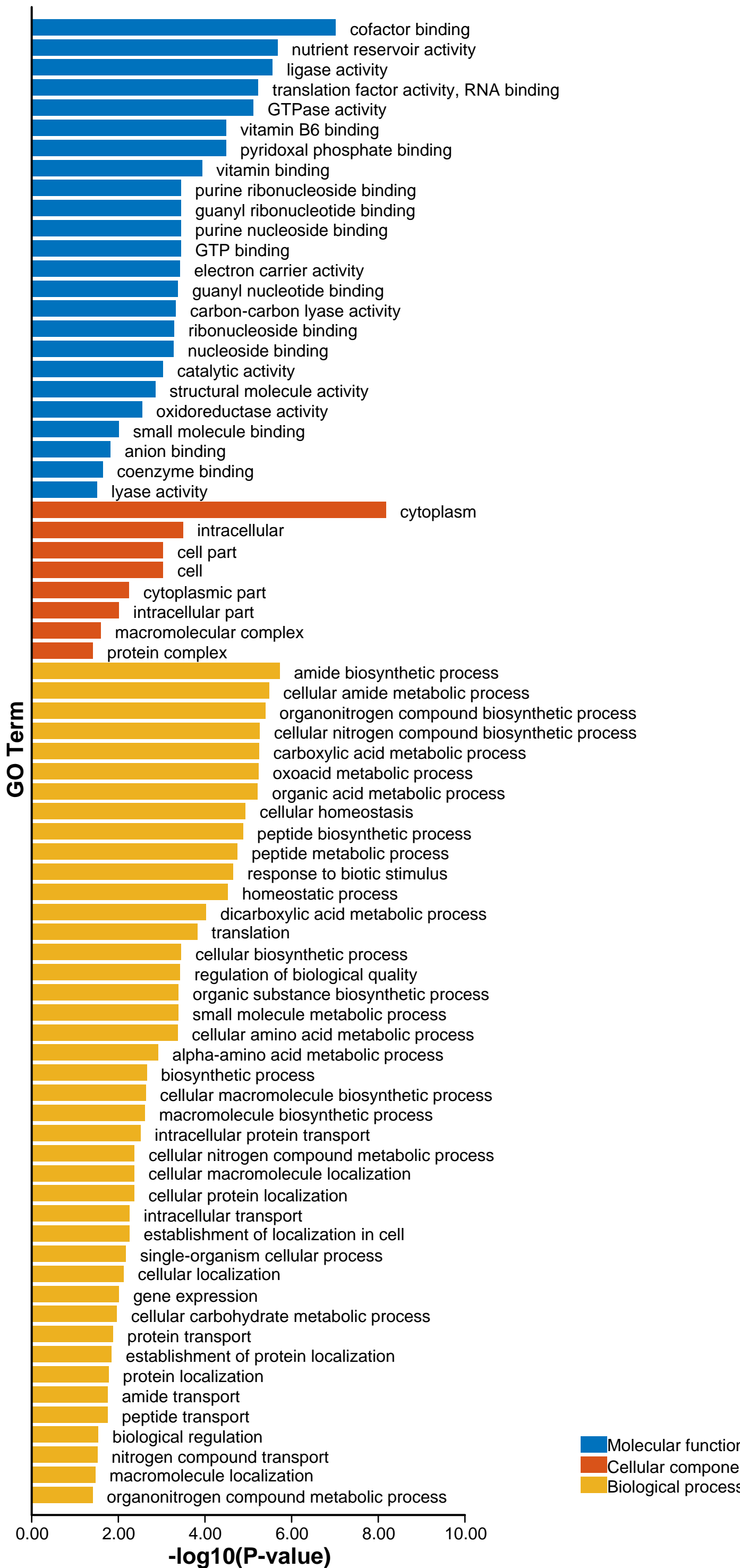

Supplement: Supplementary file 5 — Additional file 5: Fig. S5. The gene ontology enrichment of biased homeologs in the root sub-genome B. [file 13007_2022_840_MOESM5_ESM.pdf]
